# Multi-shot gradient-echo planar imaging sequence in non-contrast coronary magnetic resonance angiography

**DOI:** 10.3389/fcvm.2025.1496853

**Published:** 2025-06-02

**Authors:** Jiajia Zhu, Wenjing Li, Guangming Lu, Dongsheng Jin, Qiuju Hu, Yong Yuan, Song Luo, Yane Zhao

**Affiliations:** ^1^ Department of Radiology, Geriatric Hospital of Nanjing Medical University, Nanjing, China; ^2^ Department of Radiology, Jinling Hospital Affiliated to Nanjing University, Nanjing, China

**Keywords:** magnetic resonance imaging, echo-plane imaging, image quality, diagnostic efficiency, coronary artery

## Abstract

**Objective:**

To explore the feasibility of multi-shot gradient-echo planar imaging (MSG-EPI) sequence in non-enhanced coronary artery magnetic resonance angiography (CMRA).

**Methods:**

Patients undergoing CMRA in the Geriatric Hospital of Nanjing Medical University from November 2023 to May 2024 were included. We compared MSG-EPI and three-dimensional balanced turbo field echo (3D BTFE) sequence in acquisition time, subjective image score, signal-to-noise ratio (SNR) and contrast-to-noise ratio (CNR). With CTA as the reference standard, the linear weighted kappa and compared chi-square Mcnemar test were used to evaluate the diagnostic efficacy of both sequences for coronary artery diseases (CADs). The scale for the kappa coefficients was interpreted as follows: <0.2 = poor, 0.2–0.4 = fair, 0.4–0.6 = moderate, 0.6–0.8 = substantial, and >0.8 = excellent.

**Result:**

Seventy-two patients (33 males; mean age 54.5 ± 14.7 years old, range from 18 to 79 years old) were enrolled. MSG-EPI had a significantly shorter acquisition time than 3D BTFE (17.21 ± 1.08 s vs. 558.10 ± 102.90 s, *P* < 0.001). No significant differences in subjective scores were found between sequences for the proximal and middle segment of RCA, LM, the proximal segment of LAD and LCX (*P* = 0.168, 0.097, 0.126, 0.065, 0.062, respectively). SNR evaluations revealed no significant differences in the proximal and middle segment of RCA and LM segment (*P* = 0.119, 0.105, 0.237, respectively). However, in coronary artery segment analysis, the CNR was significantly higher in 3D BTFE compared to MSG-EPI (*P* all <0.05). The kappa values for MSG-EPI and 3D BTFE in assessing stenosis were 0.785 and 0.814, respectively. The sensitivity, specificity, positive predictive value (PPV), negative predictive value (NPV), and accuracy of MSG-EPI were 86.7%, 83.3%, 76.5%, 90.9%, and 84.6%, respectively. The area under the curve (AUC) of MSF-EPI and 3D BTFE for CADs diagnosis was 0.850 (0.699–0.944) and 0.879 (0.735–0.961), respectively (*P* = 0.543).

**Conclusion:**

MSG-EPI sequence could significantly shorten the acquisition time and provide sufficient image quality for CADs evaluation in non-enhanced CMRA.

## 1 Introduction

Coronary artery diseases (CADs) have a high incidence, early screening and accurate diagnosis of CADs are crucial for disease risk stratification and clinical management (1). Catheter-based x-ray coronary angiography is the current gold standard for the diagnosis of significant CAD, but it is an invasive procedure (2). Non-enhanced coronary magnetic resonance angiography (CMRA) is an emerging technique for assessing the morphology and function of coronary arteries without ionizing radiation or contrast media which is more suitable for children, pregnant women, and patients with contrast media allergy (3). Non-enhanced CMRA not only eliminates the risk of further kidney damage by injecting contrast media, but also avoids calcium-induced blanking artifacts. In fact, in patients with moderate-to-severe calcification, CMRA performs better than computed tomography angiography (CTA) in detecting significant stenosis (1). Moreover, CMRA has made a significant contribution to understanding the pathophysiology of CADs (4, 5), and its clinical value in the evaluation of CADs has been widely recognized (6, 7).

The three-dimensional balanced turbo-field-echo (3D BTFE) sequence is a commonly used fully balanced steady state coherent imaging pulse sequence using a very short repetition time, designed to provide bright blood images with high vessel-to-background contrast compared to other gradient echo techniques without increasing the acquisition time (8, 9). 3D BTFE sequence has been widely used in CMRA and has high diagnostic accuracy in CADs, but its clinical application is limited by the long scanning time (10). Echo-planar imaging (EPI) technique is well-known for its rapid image acquisition speed of 100 ms per slice (11, 12). However, it has limitations in terms of spatial resolution and susceptibility to off-resonance artifacts. The multi-shot gradient-echo planar imaging (MSG-EPI) sequence is an innovative rapid scanning technology that combines the advantages of gradient echo imaging and EPI, enabling the acquisition of multiple echoes with a single radiofrequency (RF) excitation (13, 14).

Previous studies have investigated the feasibility using MSG-EPI on 3.0T scanners in vascular imaging, such as thoracic aorta and renal artery, and have shown excellent performance (15, 16). Iyama et al. (17) have confirmed the feasibility of MSG-EPI sequence in non-enhanced CMRA in healthy volunteers. Yu et al. (18) compared MSG-EPI sequence and fast gradient recalled echo sequence, and found that the image quality of MSG-EPI sequence was superior to fast gradient recalled echo sequence. Currently, the image quality of MSG-EPI sequence has been evaluated, but its diagnostic ability in coronary artery stenosis has not been evaluated.

The purpose of this study was to evaluate the feasibility of MSG-EPI sequence in non-enhanced CMRA and to explore a new, rapid and non-invasive examination method for coronary artery assessment.

## 2 Methods

### 2.1 Patient population

**Figure 1:**
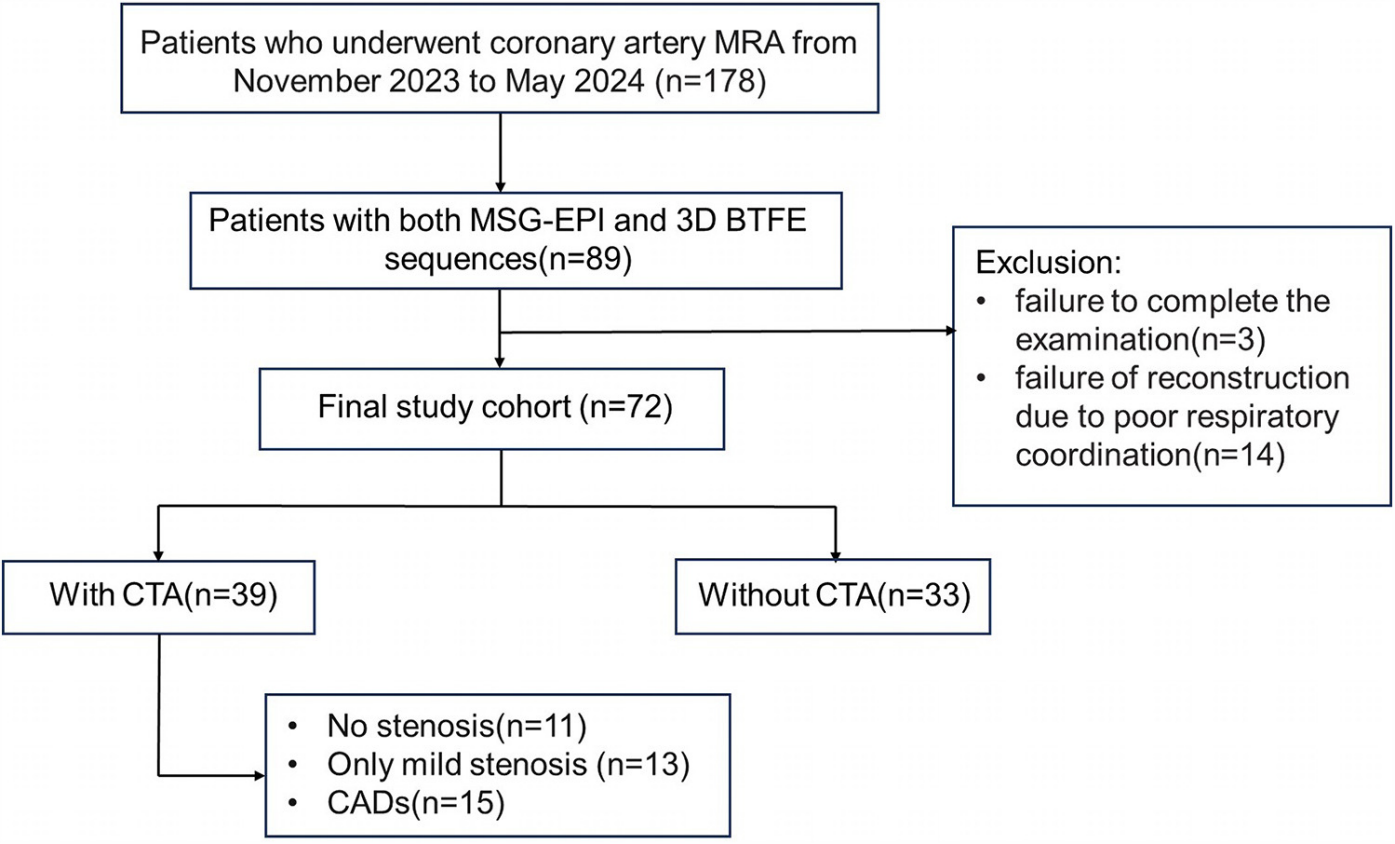
Flowchart of patient enrollment.

We retrospectively included the medical records of patients who received CMRA from November 2023 to May 2024. All procedures were performed in compliance with relevant laws and institutional guidelines. This research has been approved by the Geriatric Hospital of Nanjing Medical University (approval number 240011). Written informed consent was obtained from all subjects before the scan. The inclusion criteria were as follows: (1) MSG-EPI and 3D BTFE sequences were performed; (2) body mass index (BMI) < 28 kg/m^2^; (3) resting heart rate <80 beats/minute; patients with a heart rate of ≥ 80 beats/minute took *β*-blockers orally to lower their heart rate below 80 beats/minute; (4) no severe arrhythmia. Exclusion criteria: (1) incomplete image; (2) coronary reconstruction could not be performed, primarily due to severe artifacts. The flowchart of patient enrollment is shown in Figure 1.

### 2.2 CMRA and CTA protocol

#### 2.2.1 CMRA protocol

The 3D BTFE sequence was acquired on a 3.0T MRI scanner (Elition, Philips Healthcare, Best, the Netherlands). Breathing training was required for all patients. The retrospective ECG-triggered cine sequence was performed in the 4-chamber heart plane to determine the quiescent period window by the minimal motion of the right coronary artery (RCA). The visual evaluation was used to determine the trigger delay time and acquisition duration of every patient, freezing respiratory movement by using diaphragmatic navigation. The navigation bar was placed on the right diaphragm with lung tissue in the upper 1/3 and liver tissue in the lower 2/3. The detailed parameters of coronal 3D BTFE were as follows: repetition time/echo time = 3.1/1.56 ms, flip angle = 70°, T2 preparation time = 40 ms, field of view = 380 × 380 mm^2^, acquired voxel size = 1.4 × 1.4 × 1.4 mm^3^, slices = 330. The detailed parameters of single breath-holding MSG-EPI were as follows: repetition time/echo time = 13/6.0 ms, flip angle = 20°, field of view = 300 × 300 mm^2^, acquired spatial resolution = 1.56 × 1.95 × 3 mm^3^, acquired voxel size = 1.56 × 1.95 × 3 mm^3^, turbo field echo (TFE) factor = 18, EPI factor = 9, slices = 70.

#### 2.2.2 CTA protocol

CTA was performed on a dual-source Force CT scanner (SOMATOM Force, Siemens Healthineers, Forcheim, Germany). Scanning parameters for CTA were as follows: retrospective electrocardiogram-gated dual-source helical scan, collimation of 192 × 2 × 0.6 mm, gantry rotation time of 0.25 s, tube voltage of 120 kV, automated tube current modulation. The best diastolic phase was reconstructed using a moderately sharp vascular convolution kernel (Body vascular 36), with a slice thickness of 0.75 mm and a slice increment of 0.5 mm. The matrix size was set at 512 × 512.

### 2.3 Image quality evaluation

Curved reconstruction of the coronary artery was performed on the IntelliSpace Portal (Philips Healthcare, version 7.0). Nine coronary segments were evaluated in each individual: the left main coronary artery (LM), the proximal, middle and distal segments of the left anterior descending coronary artery (LAD) and RCA, the proximal and distal segments of the left circumflex coronary artery (LCX).

#### 2.3.1 Subjective image analysis

**Figure 2:**
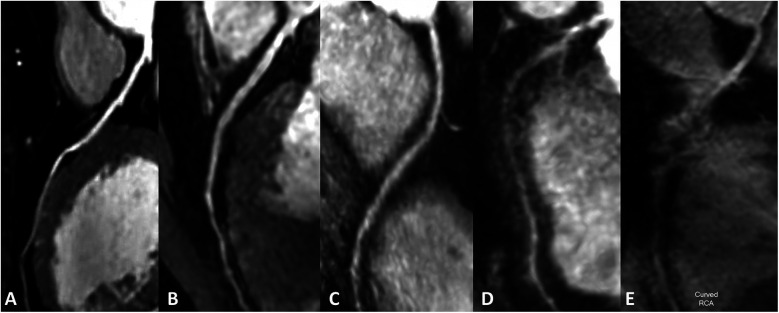
Subjective rating scale. **(A–E)** Represented images on a five-point scale. **(A)** Excellent, scored 5 (clear delineation of coronary arteries); **(B)** good, scored 4 (adequate coronary artery imaging, with diagnostic quality images); **(C)** general, scored 3 (coronary artery visible, but low diagnostic confidence); **(D)** poor, scored 2 poor (coronary artery display is almost invisible or image noise is severe); **(E)** very poor, scored 1 (coronary arteries are not visible).

Two experienced radiologists (JZ and WL, each with five years of diagnostic experience) independently assessed the images in a blinded manner. The image quality was evaluated using a 5-point scale (10, 19): 1, very poor (coronary arteries are not visible); 2, poor (coronary artery display is almost invisible or image noise is severe); 3, general (coronary artery visible, but low diagnostic confidence); 4, good (adequate coronary artery imaging, with diagnostic quality images); 5. Excellent (clear delineation of coronary arteries). Image score ≥3 points can meet the diagnostic requirements. An example image of each score is shown in Figure 2.

#### 2.3.2 Objective image quality

**Figure 3:**
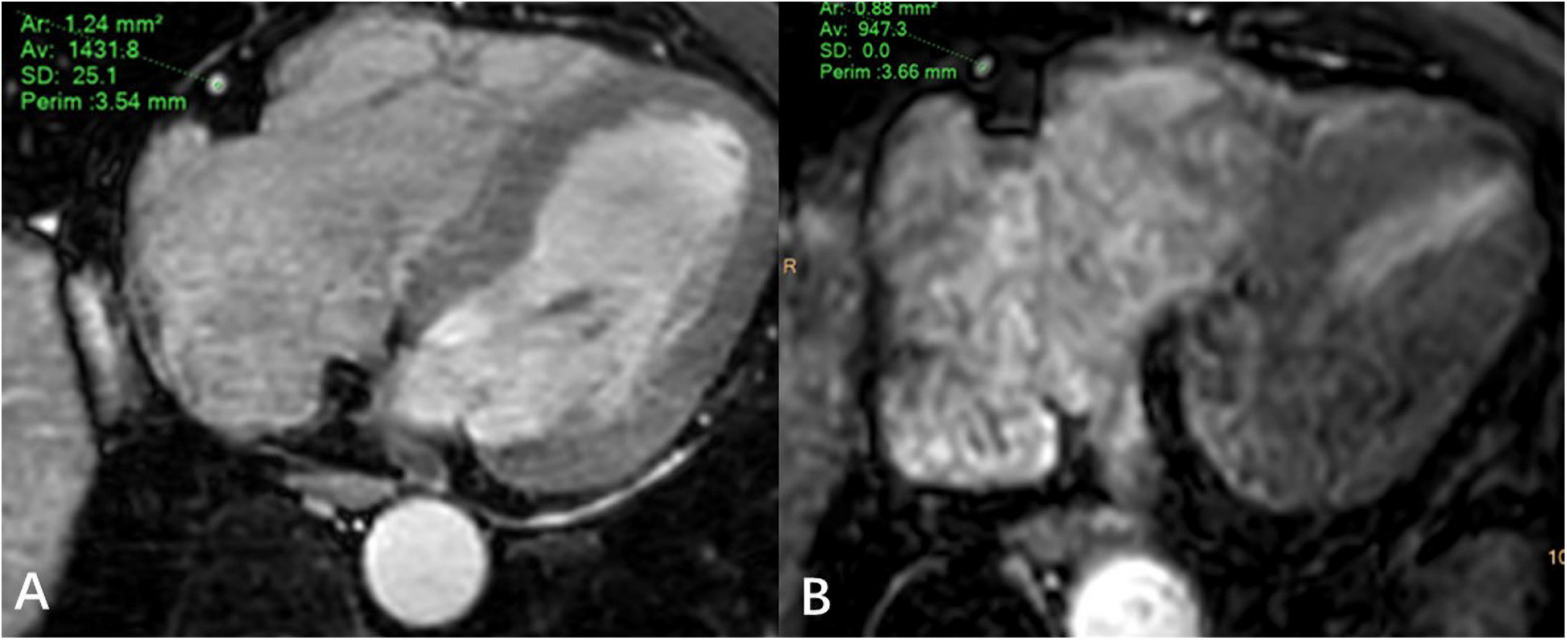
Regions of interest example diagram in axial MSG-EPI **(A)** and 3D BTFE **(B).**

JZ and WL draw regions of interest in the axial MSG-EPI and 3D BTFE sequences. The regions of interest were slightly smaller than the lumen size, and vessels, artifacts, and stenosis areas should be avoided as much as possible (Figure 3). Signal intensity (SI) and standard deviation (SD) of coronary artery segments and myocardium were measured. The signal-to-noise ratio (SNR) and contrast-to-noise ratio (CNR) of each segment of the coronary artery were calculated as follows: SNR = SI_coronary artery_/SD_myocardium_; CNR = (SI_coronary artery_ − SI_myocardium_)/SD_myocardium_. In order to minimize the deviation of a single measurement, measurements were made three times and the average value was calculated.

### 2.4 Diagnostic accuracy

Coronary artery stenosis was classified into four grades: non-stenosis, mild stenosis (25%–49% stenosis), moderate stenosis (50%–69% stenosis), and severe stenosis (≥70% stenosis). Each segment was diagnosed in MSG-EPI sequence and 3D BTFE sequence respectively, and the diagnostic consistency of the two sequences relative to CTA was analyzed.

Clinically significant CADs was defined as a diameter reduction of at least 50% (19). According to at least one vessel of coronary artery lumen stenosis ≥50%, coronary artery stenosis was divided into two categories to determine whether CADs was present. Using CTA as the reference standard, the diagnostic performance of the MSG-EPI sequence and 3D BTFE sequence was evaluated respectively. Disagreements between the two observers (JZ and WL) were settled by YZ, a radiologist with more than 20 years of experience.

### 2.5 Statistical analysis

All statistical analyses were performed using the SPSS software package (version 27.0, Chicago, IL, USA) and MedCalc (version 20.0, Mariakierke, Belgium). Paired t-test or Wilcoxon sign rank test was used to compare acquisition time, subjective and objective image quality of MSG-EPI and 3D BTFE sequences.

Using CTA as the reference standard, linear weighted kappa method was used to evaluate the consistency of MSG-EPI sequence and 3D BTFE sequence in predicting each coronary artery segment stenosis. The diagnostic performance of the MSG-EPI sequence and 3D BTFE sequence for indicating the presence of CADs was evaluated by the compared chi-square Mcnemar test. Receiver operating characteristic was used to evaluate the diagnostic efficiency of CADs in 3D BTFE sequence and MSG-EPI sequence. The area under the curve (AUC) and 95% confidence interval were calculated, and the difference of AUC was compared by Delong test. The scale for the kappa coefficients was interpreted as follows: <0.2 = poor, 0.2–0.4 = fair, 0.4–0.6 = moderate, 0.6–0.8 = substantial, and >0.8 = excellent. *P* values of <0.05 were determined to be of statistical significance.

## 3 Results

### 3.1 Patients' characteristic

**Figure 4:**
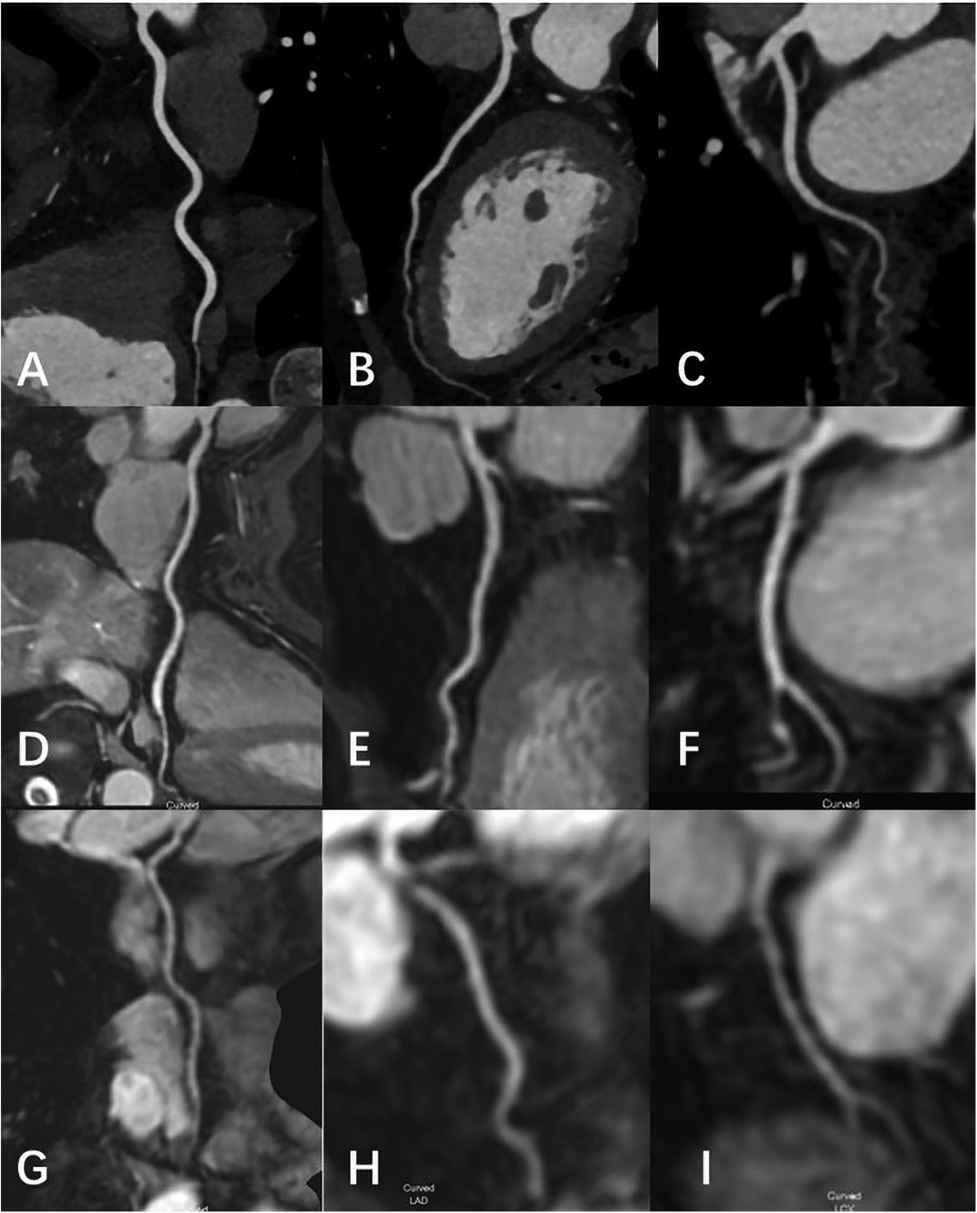
Female, 69 Y, with chest pain. **(A–C)** The RCA, LAD, LCX shown in coronary CTA, coronary artery stenosis was not observed; **(D-F)** the RCA, LAD, LCX shown in 3D-BTFE sequence, the lumen edges were sharp, the coronary arteries were clearly delineated, and the subjective images scored 5; **(G–I)** the RCA, LAD, LCX shown in MSG-EPI sequence, and the subjective images scored 4.

**Figure 5:**
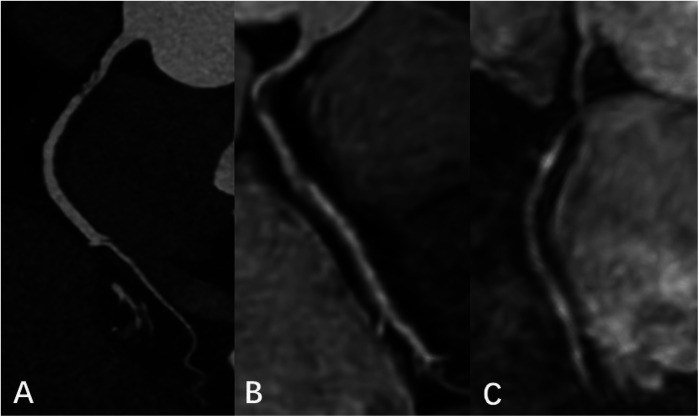
Male, 66 Y, with moderate stenosis of RCA. CTA **(A)** showed mixed plaque in the proximal segment of RCA with moderate lumen stenosis. 3D BTFE **(B)** and MSG-EPI **(C)** also showed local moderate stenosis of the RCA proximal segment.

**Figure 6:**
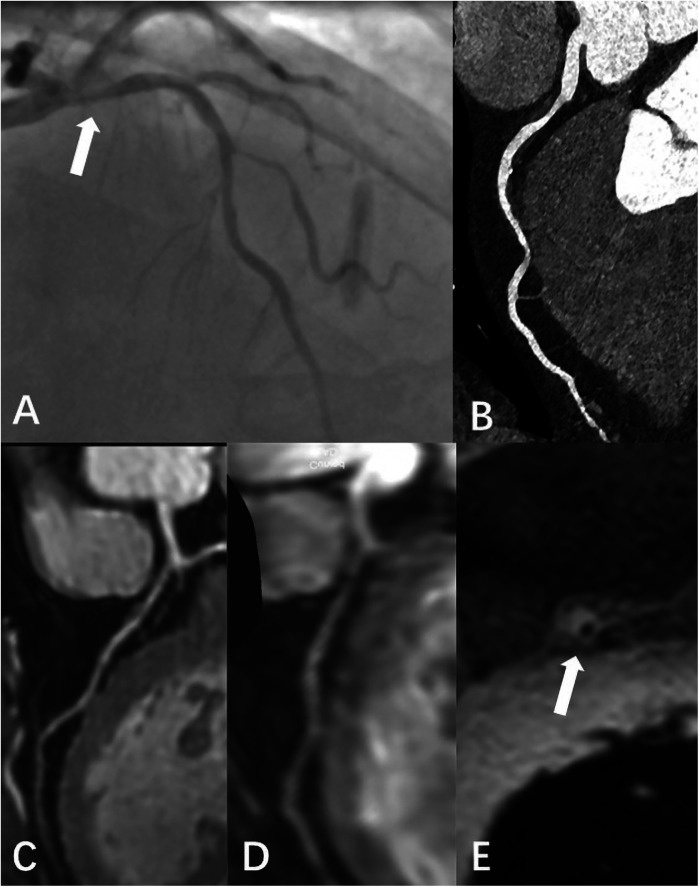
Male, 59 Y, with chest pain. **(A)** Catheter-based x-ray coronary angiography indicated a 70% stenosis at the origin of the LAD. **(B)** CTA revealed non-calcified plaque surrounding the lumen at the proximal LAD, with moderate stenosis. **(C)** 3D BTFE showed a localized signal reduction in the proximal vessel wall. **(D)** MSG-EPI indicated moderate stenosis in the proximal lumen. **(E)** Axial T1-weighted fat-suppressed black-blood sequence suggested the presence of a high-signal plaque surrounding the vessel wall.

Eighty-nine patients underwent both MSG-EPI sequence and 3D BTFE sequence from November 2023 to May 2024. Three cases were excluded due to failure to complete the examination. Fourteen cases were excluded due to severe artifacts in the MSG-EPI sequence. Successful reconstruction was achieved in nearly all instances with 3D BTFE sequence, although there were some cases in which the subjective score of the vessel was less than 3. Finally, 72 patients (33 males; mean age 54.5 ± 14.7 years old, range from 18 to 79 years old) were included in the study. The clinical information of patients is shown in Table 1. Typical cases are shown in Figures 4–6.

**Table 1:** Patient characteristics.

Characteristic	All patients (*n* = 72)	Patients who underwent CTA (*n* = 39)
Male	33 (45.8)	18 (46.2)
Age (mean ± SD, year)	54.5 ± 14.7	60.9 ± 8.9
BMI (mean ± SD, kg/m^2^)	22.4 ± 3.3	23.3 ± 1.9
Coronary risk factor		
Hypertension	12 (16.7)	7 (17.9)
Dyslipidemia	14 (19.4)	9 (23.1)
Diabetes	11 (15.2)	6 (15.4)
Current smoker	17 (23.6)	11 (28.2)
Family history of CADs	14 (19.4)	9 (23.1)
Statin use	7 (9.7)	7 (17.9)
Underwent CTA within 1 month	39 (54.2)	
No stenosis	11 (28.2)	
Only mild stenosis (25%–49% stenosis)	13 (33.3)	
CADs (≥50% stenosis in at least one blood vessel)	15 (38.5)	

Except where indicated, data were numbers of patients (percentages). BMI, body mass index; CAD, coronary artery disease.

### 3.2 Acquisition time

The acquisition time of MSG-EPI sequence was significantly shortened compared with that of 3D BTFE sequence (17.2 ± 1.1 s vs. 558.1 ± 102.9 s, *P* < 0.001).

### 3.3 Subjective image analysis

At the level of coronary artery segments, the subjective scores of the RCA distal segment, LAD middle and distal segment and LCX distal segment in 3D BTFE sequence were significantly higher than those in MSG-EPI sequence (*P* = 0.002, <0.001, <0.001, <0.001, respectively). There were no significant differences in the proximal and middle segment of RCA, LM, the proximal segment of LAD and LCX between MSG-EPI sequence and 3D BTFE sequence (*P* = 0.168, 0.097, 0.126, 0.065, 0.062, respectively). At the level of coronary artery vascular, the subjective scores of LAD and LCX in 3D BTFE sequence were significantly higher than those in MSG-EPI sequence (*P* values all <0.001). There was no significant difference between MSG-EPI sequence and 3D BTFE sequence in RCA (*P* = 0.061). At the individual level, the subjective scores of the MSG-EPI sequence were lower than those of 3D BTFE sequence [4 (4, 4.8) vs. 4 (4, 5), *P* < 0.01]. Details are shown in Table 2.

**Table 2:** Coronary artery subjective scores in 3D BTFE and MSG-EPI sequences.

Analysis basis	3D BTFE	MSG -EPI	*P* value
In segment			
RCA p	5 (4, 5)	5 (4, 5)	0.168
RCA m	5 (4, 5)	4 (4, 5)	0.097
RCA d	4 (4, 5)	4 (3.3,4)	0.002*
LM	5 (4, 5)	5 (4, 5)	0.126
LAD p	5 (4, 5)	4.5 (4, 5)	0.065
LAD m	4 (4, 5)	4 (4, 4)	0.001*
LAD d	4 (4, 4.8)	4 (3, 4)	0.001*
LCX p	4 (4, 5)	4 (4, 4)	0.062
LCX d	4 (4, 4)	4 (3, 4)	0.001*
In vascular			
RCA	5 (4, 5)	4 (4, 5)	0.061
LAD	5 (4, 5)	4 (4, 5)	<0.001*
LCX	4 (4, 5)	4 (4, 4)	<0.001*
In individual	4 (4, 5)	4 (4, 4.8)	<0.001*

3D BTFE, three-dimensional balanced turbo field echo; MSG-EPI, multi-shot gradient-echo planar imaging; RCA, right coronary artery; LM, left main artery; LAD, left anterior descending; LCX, left circumflex; p, m and d represent the proximal, middle and distal segments of the coronary artery, respectively. Data were reported as median (the first quartile, the third quartile).

* Represented significant difference.

### 3.4 Objective image quality

In the evaluation of SNR in each coronary artery segment, there was no difference between 3D BTFE sequence and MSG-EPI sequence in the proximal and middle segment of RCA and LM segment (*P* = 0.119, 0.105, 0.237, respectively), while in the distal segment of RCA, three segments of LAD and two segments of LCX, the SNRs of 3D BTFE sequence were significantly higher than those of the MSG-EPI sequence (*P* = 0.043, 0.017, 0.003, <0.001, <0.001, <0.001, respectively). In the evaluation of CNR, each segment of the 3D BTFE sequence was significantly higher than that of MSG-EPI sequence (*P* all <0.001). Details are shown in Table 3.

**Table 3:** SNR and CNR in 3D BTFE and MSG-EPI sequences.

Parameters	RCA p	RCA m	RCA d	LM	LAD p	LAD m	LAD d	LCX p	LCX d
SNR									
3D BTFE	28.3 (22.9, 36.4)	27.1 (21.9, 35.5)	27.1 (21.1, 33.3)	27.7 (22.0,36.4)	26.5 (22.0, 34.6)	23.0 (18.4, 32.1)	21.0 (16.1,28.4)	27.5 (23.0, 37.0)	25.5 (18.1, 35.1)
MSG-EPI	26.8 (18.3, 35.2)	24.5 (18.2,32.5)	22.5 (16.3,32.3)	26.5 (22.1,33.5)	24.0 (16.0, 30.7)	19.2 (13.4, 25.9)	15.5 (11.2,21.4)	22.3 (16.3, 28.9)	19.6 (13.9, 24.8)
*P* value	0.119	0.105	0.043 *	0.237	0.017 *	0.003 *	<0.001 *	<0.001 *	<0.001 *
CNR									
3D BTFE	12.9 (10.0, 19.3)	10.9 (7.1,17.4)	11.2 (5.9,16.1)	12.8 (8.6, 19.6)	11.6 (7.9,17.3)	6.9 (4.1, 12.3)	5.6 (2.2, 14.2)	12.1 (7.3, 18.0)	9.5 (3.6, 17.5)
MSG-EPI	6.7 (1.8, 12.6)	6.6 (3.2,10.9)	5.1 (2.4,9.3)	8.8 (4.1, 11.3)	5.0 (0.6, 8.9)	0.1(-3.3, 4.5)	-1.8(-7.4, 1.4)	5.6 (1.0, 9.4)	1.8(-1.8, 6.2)
*P* value	<0.001 *	<0.001 *	<0.001 *	<0.001 *	<0.001 *	<0.001 *	<0.001 *	<0.001 *	<0.001 *

3D BTFE, three-dimensional balanced turbo field echo; MSG-EPI, multi-shot gradient-echo planar imaging; RCA, right coronary artery; LM, left main artery; LAD, left anterior descending; LCX, left circumflex; p, m and d represent the proximal, middle and distal segments of the coronary artery, respectively. SNR, signal-to-noise ratio; CNR, contrast-to-noise ratio. Data were reported as median (the first quartile, the third quartile).

* Represented significant difference.

### 3.5 Diagnostic accuracy

Among 72 patients, 39 patients underwent CTA within one month. Among the 39 patients, 11 cases had no stenosis, 13 cases had only mild stenosis, 15 cases had CADs, and 5 cases had severe stenosis as confirmed by CTA. Using CTA as the reference standard to evaluate each coronary artery segment, the kappa value of 3D BTFE and MSG-EPI were 0.814 and 0.785, respectively.

**Figure 7:**
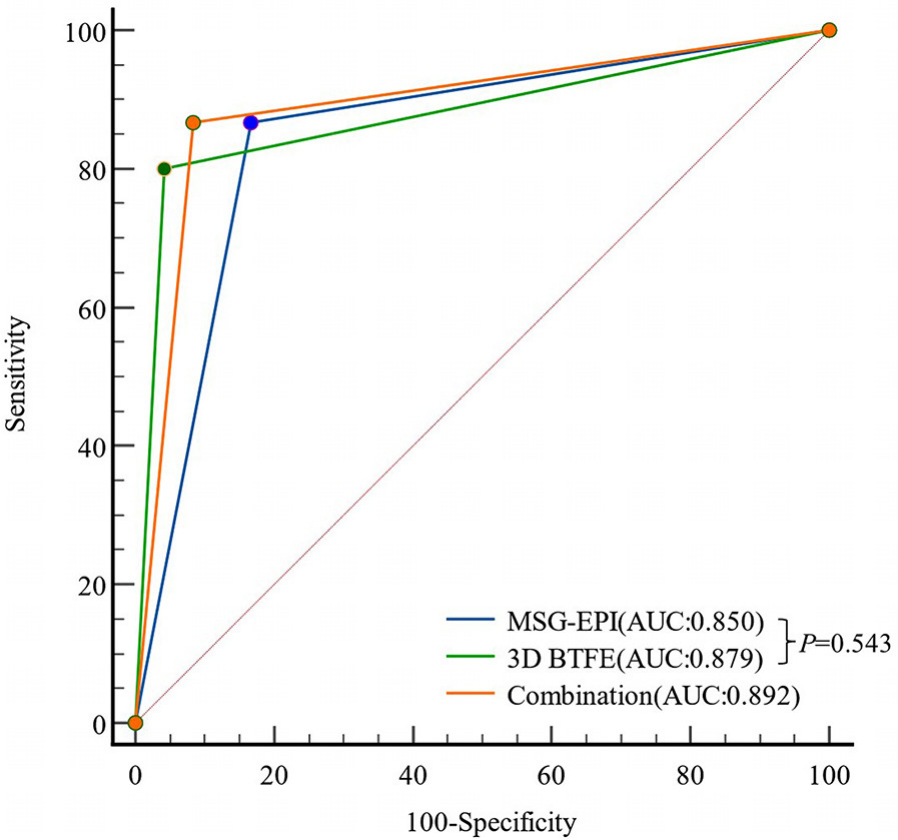
Receiver operating characteristic curves of MSG-EPI and 3D BTFE for the diagnosis of CADs.

In chi-square Mcnemar test, there was no significant difference between CTA and 3D BTFE sequence, CTA and MSG-EPI sequence in the diagnostic efficiency of CADs (*P* = 0.625 and 0.687). The sensitivity, specificity, positive predictive value (PPV), negative predictive value (NPV) and accuracy of MSG-EPI sequence, 3D BTFE sequence and the combination of these two sequences in the diagnosis of CADs are shown in Table 4. The comparison of MSG-EPI sequence and 3D BTFE sequence in evaluating patients with CADs showed that the kappa value of 3D BTFE sequence relative to CTA was 0.778, the kappa value of MSG-EPI sequence was 0.683, and the AUC was 0.879 (0.735–0.961) vs. 0.850 (0.699–0.944), *P* = 0.543 (Figure 7).

**Table 4:** MSG-EPI sequence and 3D BTFE sequence in the diagnosis of CADs.

Sequences	Results				Sensitivity (%)	Specificity (%)	PPV (%)	NPV (%)	Accuracy (%)	Kappa	AUC (95%CI)
	TP	FP	FN	TN							
MSG-EPI	13	4	2	20	86.7	83.3	76.5	90.9	84.6	0.683	0.850 (0.699∼0.944)
3D BTFE	12	1	3	23	80.0	95.8	92.3	88.5	89.7	0.778	0.879 (0.735∼0.961)
MSG-EPI & 3D BTFE	13	2	2	22	86.7	91.7	86.7	91.7	89.7	0.783	0.892 (0.750∼0.968)

3D BTFE, three-dimensional balanced turbo field echo; MSG-EPI, multi-shot gradient-echo planar imaging; TP, true positive; FP, false positive; FN, false negative; TN, true negative; PPV, positive percent agreement; NPV, negative percent agreement; AUC, area under the curve; 95%CI, 95% confidence interval; MSG-EPI & 3D BTFE, represents the combination of MSG-EPI sequence and 3D BTFE sequence.

## 4 Discussion

In this study, the scanning time of MSG-EPI sequences was significantly reduced by 96.9% compared with 3D BTFE sequences (17.2 ± 1.1 s vs. 558.1 ± 102.9 s, *P* < 0.001). Despite the significantly shorter scanning time, the image quality of MSG-EPI was comparable to that of 3D BTFE, with no significant differences in the scores for the proximal and middle coronary artery segments. While the diagnostic specificity and accuracy of MSG-EPI in the diagnosis of CADs were slightly lower than those of 3D BTFE, MSG-EPI demonstrated higher sensitivity. The combination of both methods resulted in an increased AUC and improved diagnostic efficiency.

Most scanners used for CMRA were 1.5T in the early 2010s. In 1.5T MRI, balanced steady-state free precession imaging (bSSFP) is the most commonly used sequence. However, the applicability of bSSFP in 3T MRI is limited due to the inhomogeneity of B0 and B1 fields, RF pulse-induced dielectric effects, and power deposition in the human body (3). But in recent CMRA studies, more studies have been performed at 3.0T. Studies have shown that when performed with the same sequence, 3.0T coronary MRA was not inferior to 1.5T coronary MRA both in image quality and diagnostic accuracy for the detection of coronary stenosis (4, 17, 20, 21).

Our findings align with previous studies, which have consistently shown that MSG-EPI scanning times are much shorter than those of bSSFP or TFE sequences (15–17). However, MSG-EPI has a drawback in that it requires breath-holding, which poses challenges for clinical application, especially given the small vessel size, interference from fat and myocardium, vessel tortuosity, and physiological motion (22, 23). In this study, 14 patients were excluded due to poor breath-holding coordination, resulting in a lower reconstruction success rate for the MSG-EPI sequence (83.7%) compared to the 3D BTFE sequence. To increase the success rate of MSG-EPI examinations, it is recommended to provide patients with breathing training prior to the examination. However, the scanning time of MSG-EPI in this study was reduced by 30.6% (17.2 ± 1.1 s vs. 24.7 ± 2.5 s) compared to previous reports (17), which was more friendly to patients with respiratory diseases. Additionally, the MSG-EPI sequence can enhance diagnostic confidence in cases where 3D BTFE sequence results are uncertain, potentially eliminating the need for prolonged repeated scans.

In this study, it was found that there was no significant difference in subjective scores and SNR between MSG-EPI sequences and 3D BTFE sequences in the proximal and middle segments of coronary artery, indicating that MSG-EPI provided comparable diagnostic detail in critical regions of the coronary anatomy. Previous studies have also highlighted the potential of MSG-EPI to maintain high spatial resolution and SNR, which were crucial for accurate coronary artery evaluation (17, 18, 24). In terms of CNR, each segment of coronary artery in MSG-EPI sequence was significantly lower than that of 3D BTFE. This was likely due to the sensitivity of MSG-EPI to blood flow and heart movement and the T2^∗^ decay may introduce blurring in the images.

Previous studies in coronary arteries on MSG-EPI sequence were mostly focused on the evaluation of image quality, without evaluating its diagnostic ability. In this study, CTA was used as the standard to evaluate the degree of stenosis in each segment of coronary artery, and it was found that MSG-EPI sequence and 3D BTFE sequence had good diagnostic consistency compared to CCTA (kappa values = 0.683 vs. 0.778). Furthermore, in the diagnosis of CADs, there was no significant difference between MSG-EPI and 3D BTFE in the diagnostic efficacy of CADs. It was confirmed that MSG-EPI sequence had the similar diagnostic value in CADs diagnosis as 3D BTFE sequence, and affirmed the ability of MSG-EPI sequence in the diagnosis of coronary artery stenosis. Moreover, we found that the ability to diagnose CADs was improved after combining MSG-EPI sequence and 3D BTFE sequence. Our study indicated that MSG-EPI sequence based on 3T MRI system could provide sufficient image quality and SNR in whole-heart coronary MRA to evaluate coronary artery morphology and stenosis since the SNR was proportional to the static field strength (24).

This study has some limitations. The diagnostic accuracy was primarily based on CTA due to the small sample size of Catheter-based x-ray coronary angiography. Additionally, the sample size of patients with stenosis confirmed by CTA was relatively small, which contributed to the insufficient diagnostic accuracy of MSG-EPI. Future work will involve increasing the sample size and conducting stratified analyses based on the degree of stenosis and anatomical regions to further validate the feasibility and diagnostic efficacy of this technique. The large sample data can be utilized to optimize images using artificial intelligence algorithms. Finally, this study did not evaluate imaging parameters such as EPI factor and TFE factor on image quality. Future research should focus on exploring the effects of different systems and parameters on the MSG-EPI sequence to find the best parameters and optimizing its performance.

## 5 Conclusion

MSG-EPI sequence could significantly shorten the acquisition time and provide sufficient image quality for CADs evaluation in non-enhanced CMRA.

## Data Availability

The original contributions presented in the study are included in the article/Supplementary Material, further inquiries can be directed to the corresponding authors.
